# Relationship between the expression of oestrogen receptor and progesterone receptor and ^18^F-FDG uptake in endometrial cancer

**DOI:** 10.18632/aging.103352

**Published:** 2020-07-08

**Authors:** Chunhua Wu, Ruohua Chen, Lian Xu, Yumei Chen, Yining Wang, Gan Huang, Jianjun Liu

**Affiliations:** 1 Department of Nuclear Medicine, Ren Ji Hospital, School of Medicine, Shanghai Jiao Tong University, Shanghai, China; 2 Department of Ultrasound, Ren Ji Hospital, School of Medicine, Shanghai Jiao Tong University, Shanghai, China

**Keywords:** endometrial cancer, ER, PR, PET/CT, SUVmax

## Abstract

Background: Progestogens have been widely used for the treatment of inoperable endometrial cancer or younger patients with endometrial cancer. Identifying markers that are predictive of a response to progestogens is critical for successful therapy. Molecular imaging with ^18^F^-^fluorodeoxyglucose positron emission tomography (^18^F-FDG PET) can provide metabolic phenotypic information of many malignancies. We investigated whether estrogen receptor (ER)/progestogen receptor (PR) status is correlated with ^18^F-FDG uptake, and whether ^18^F-FDG PET/CT could be useful for predicting ER/PR status in endometrial cancer.

Results: Endometrial cancers in the ER-positive group had lower SUVmax than those in the ER-negative group (12.3 ± 6.2 vs. 19.9 ± 6.6, respectively; P = 0.003). Endometrial cancers in the PR-positive group also had lower SUVmax than those in the PR-negative group (12.4 ± 6.2 vs. 20.0 ± 6.9, respectively; P = 0.005). Multivariate analysis indicated that SUVmax and tumour differentiation grade were significantly associated with both ER and PR status (P = 0.027 and P = 0.044, respectively). ER expression was predicted with an accuracy of 74.2% when a SUVmax value of 15.3 was used as a cutoff point for analysis. Similarly, PR expression was predicted with an accuracy of 74.2%, when a SUVmax value of 15.95 was used as the threshold for analysis.

Conclusion: Higher ^18^F-FDG accumulation in endometrial cancers is correlated with negative ER/PR expression. ^18^F-FDG PET/CT may be used to predict the status of ER/PR and thus aid in optimal treatment decision in endometrial cancers.

Methods: We carried out a retrospective analysis on 62 endometrial cancer patients who underwent ^18^F-FDG PET/CT before radical treatment. The maximum of standardized uptake value (SUVmax) was calculated from the ^18^F-FDG accumulation of the primary tumor. The relationship between SUVmax and ER/PR status was analyzed.

## INTRODUCTION

Endometrial cancer is one of the most frequently gynaecological malignancies worldwide [1, 2]. Radical treatment remains the main therapeutic approach for endometrial cancer [3, 4]. However, sometimes endometrial cancer is diagnosed at an advanced stage with inoperable or metastatic disease. In patients with inoperable or metastatic endometrial cancer with endometrial cancer, conservative treatment such as progestogens are widely used instead of surgery [5, 6]. However, quite a part of patients do not respond to conservative treatment [7]. Previous studies demonstrate that patients with high ER/PR expression show a higher overall response rate than that shown by patients with low ER/PR expression among patients treated with progestogens [8–10]. Therefore, in patients with endometrial cancer, it is meaningful to identify a clinicopathologic feature that is predictive of the status of estrogen receptor (ER)/progestogen receptor (PR) expression. However, thus far, there are no validated clinicopathologic characteristics to select *a priori* patients who may benefit from conservative treatment in endometrial cancer.

^18^F^-^fluorodeoxyglucose positron emission tomography (^18^F-FDG PET) is a noninvasive diagnostic tool widely used in diagnosis and staging of endometrial carcinoma [11–13]. Our previous studies suggested that ^18^F-FDG PET/CT could be useful for predicting molecular phenotype in several malignant tumors, including PD-L1 in bladder cancer [14], LDHA expression in lung cancer, and FBP1 expression in hepatocellular carcinoma [15, 16]. Many previous reports have suggested an inverse correlation between ^18^F-FDG accumulation of the primary tumour and ER/PR status in breast cancer [17–19]. However, the correlation between ^18^F-FDG accumulation and ER/PR status in endometrial cancer, and the possible underlying molecular mechanisms, are still unclear.

In the present study, we assessed whether ER/PR status of the primary tumour in endometrial cancer is correlated with ^18^F-FDG accumulation and whether ^18^F-FDG PET/CT can be useful for predicting ER/PR status in endometrial cancer. So far, our study is the first to provide data about the potential use of ^18^F-FDG PET/CT in the prediction of ER/PR status in endometrial cancer, as well as to show that ^18^F-FDG PET/CT has great effects on determining optimal treatment methods by predicting the response to progestogen treatment in endometrial cancers.

## RESULTS

### Study population

Patients’ clinicopathologic features are shown in Table 1. A total of 62 women (median age, 55.2 years; range, 29-76 years) were included in this study, of which 36 patients were already menopausal. Before ^18^F-FDG PET/CT scans, endometrial cancer was confirmed in 15 patients by hysteroscopy or colposcopy and in 45 patients by curettage. The mean time from biopsy to the scan was 16.0 days. Among the 62 cases, 49 had well- or moderately differentiated endometrial carcinoma, while 13 had poorly differentiated endometrial carcinoma. The SUVmax of endometrial cancers ranged from 2 to 33.2, with an average of 13.5. Positive ER expression was found in 83.9% (52/62) of the primary tumours, and positive PR expression was found in 85.5% (53/62) of the primary tumours.

**Table 1:** Patient characteristics (n = 62).

**Characteristics**	**No. of Patients**
**Age (y)**	
Mean ± SD	55.2±11.2
Range	29-76
**Menopause status**	
Pre	26
Post	36
Time from biopsy to scan (days)	16.0±9.1
**Biopsy method before scan**	
Hysteroscopy or colposcopy	15
Curettage	45
None	2
**Myometrial invasion**	
<50%	45
≥50%	17
**Histologic type**	
Well- or moderately differentiated	49
Poorly differentiated	13
Tumor size (cm)	2.9±1.8
**FIGO stage**	
1	56
2-4	6
**SUVmax**	
Mean ± SD	13.5±6.8
Range	2-33.2
**ER expression**	
Negative	10
Positive	52
**PR expression**	
Negative	9
Positive	53

### Correlation between SUVmax and ER/PR expression

**Figure 1:**
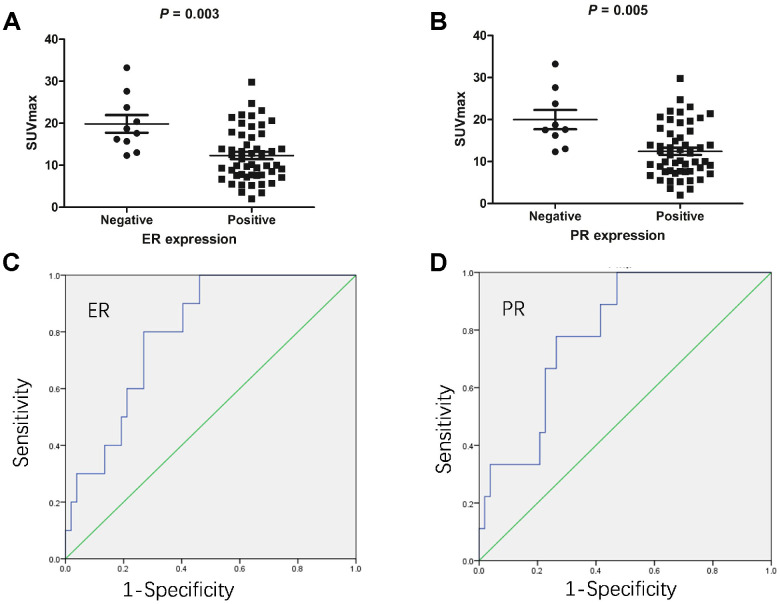
**The association between ^18^F-FDG accumulation and ER/PR status in endometrial cancers (n = 62).** (**A**) The association between ^18^F-FDG accumulation and ER status. Endometrial cancers in the ER-positive group had lower SUVmax than those in the ER-negative group (12.3 ± 6.2 vs. 19.9 ± 6.6, respectively; P = 0.003). (**B**) The association between ^18^F-FDG accumulation and PR status. Endometrial cancers in the PR-positive group also had lower SUVmax than those in the PR-negative group (12.4 ± 6.2 vs. 20.0 ± 6.9, respectively; P = 0.005). (**C**) ROC analysis of SUVmax for predicting ER status. When the cutoff threshold of SUVmax was 15.3, the sensitivity and specificity to predict ER expression were 73.1% and 80.0%, respectively. The area under curve was 0.8 (95% confidence interval: 0.679-0.921; P = 0.003). (**D**) ROC analysis of SUVmax for predicting PR status. When the cutoff threshold of SUVmax was 15.95, the sensitivity and specificity to predict PR expression were 73.6% and 77.8%, respectively. The area under curve was 0.792 (95% confidence interval: 0.663-0.992; P = 0.005).

We investigated ER/PR status by immunohistochemical analysis. In the primary tumours, we identified a negative association between SUVmax and the status of ER (Figure 1A) and PR (Figure 1B). Endometrial cancers in the ER-positive group had lower SUVmax than those in the ER-negative group (12.3 ± 6.2 vs. 19.9 ± 6.6, respectively; P = 0.003). Endometrial cancers in the PR-positive group also had lower SUVmax than those in the PR-negative group (12.4 ± 6.2 vs. 20.0 ± 6.9, respectively; P = 0.005).

We next determined the optimal SUVmax threshold for predicting ER and PR expression. ROC analysis demonstrated that the highest accuracy (74.2%) to predict ER expression was obtained when the SUVmax threshold was 15.3, resulting in area under curve of 0.8 ± 0.062. The sensitivity and specificity of this value for the prediction of ER status was found to be 73.1% (38/52) and 80% (8/10), respectively (Figure 1C). Likewise, ROC analysis also demonstrated that the highest accuracy (74.2%) to predict PR expression was obtained when the SUVmax threshold was 15.95, resulting in area under curve of 0.792 ± 0.066. Similarly, the sensitivity and specificity of this value for the prediction of PR status were found to be 73.6% (39/53) and 77.8% (7/9), respectively (Figure 1D). Taken together, these results demonstrate that SUVmax may be used to predict ER/PR status in endometrial cancer.

### Correlation between clinicopathologic characteristics and ER/PR status

Patients were separated into two groups on the basis of ER/PR status. The associations between clinicopathologic characteristics in endometrial cancers and ER/PR status were evaluated (Table 2). No significant differences in biopsy method, time from biopsy to scan, lymph node metastasis, or tumour size were observed between ER-positive and ER-negative groups. Whereas, the groups differed significantly in SUVmax, histologic type, age, menopause status, and FIGO stage (Table 2). In the multivariate analysis, SUVmax of the primary tumour and tumour differentiation grade remained significantly associated with ER status in endometrial cancer (Table 3). Similar correlations were also observed for expression of PR (Table 3).

**Table 2:** Relationship between ER/PR expression and clinicopathological characteristics in endometrial cancer (n = 62).

**Characteristics**	**Total**	**ER expression**		**P value**	**PR expression**		**P value**
		**Negative**	**Positive**		**Negative**	**Positive**	
**Age**		64.4±5.3	53.4±11.2	0.004	64.7±5.9	53.6±11.2	0.005
**Menopause status**							
Pre	26	1	25	0.025	1	25	0.043
Post	36	9	27		8	28	
**Biopsy method**							
Hysteroscopy or colposcopy	15	2	13	0.689	2	13	0.835
Curettage	45	8	37		7	38	
**Time from biopsy to scan**		15.6±8.6	16.1±9.3	0.886	18.8±9.7	15.5±9.0	0.323
**Myometrial invasion**							
<50%	45	3	42	0.001	3	42	0.004
≥50%	17	7	10		6	11	
**Lymph node metastasis**							
Absent	57	8	49	0.13	8	49	0.717
Present	5	2	3		1	4	
**Histologic type**							
Well/moderately differentiated	49	3	46	<0.001	2	47	<0.001
Poorly differentiated	13	7	6		7	6	
Tumor size (cm)		3.4±1.7	2.8±1.8	0.362	3.1±1.1	2.9±1.9	0.82
**FIGO stage**							
1	56	7	49	0.048	5	51	0.003
2-4	6	3	3		4	2	
**SUVmax**		19.9±6.6	12.3±6.2	0.003	20.0±6.9	12.4±6.2	0.005

**Table 3:** Multivariate analysis of ER/PR expression in patients with endometrial cancer.

**Predictors**	**Factor**	**Odds ratio**	**95% Confidence interval**	**P**
**ER**	Age	0.855	0.706-1.037	0.112
	Menopause status	1.412	0.016-125.322	0.88
	Tumor differentiation	0.029	0.002-0.549	0.029
	FIGO stage	7.191	0.276-187.288	0.236
	SUVmax	0.82	0.687-0.978	0.027
**PR**	Age	0.881	0.728-1.065	0.19
	Menopause	0.636	0.002-232.969	0.88
	Tumor differentiation	0.047	0.003-0.836	0.037
	FIGO stage	0.852	0.04-18.324	0.919
	SUVmax	0.808	0.656-0.994	0.044

On the basis of above two parameters including SUVmax and tumour differentiation grade, we categorized endometrial cancers into three groups to infer their potential of being ER-positive: a low-potential group (SUVmax > 15.3 and poorly differentiated), a moderate-potential group (SUVmax < 15.3 and poorly differentiated, or SUVmax > 15.3 and well- or moderately differentiated), and a high-potential group (SUVmax < 15.3 and well- or moderately differentiated). The probability of ER-positive status in these groups was 37.5% (3/8), 73.7% (14/19), and 100.0% (35/35), respectively (P < 0.001; Table 4). Similarly, based on these two parameters, we categorized endometrial cancers into three groups to infer their potential of being PR-positive: a low-potential group (SUVmax > 15.95 and poorly differentiated), a moderate-potential group (SUVmax < 15.95 and poorly differentiated, or SUVmax > 15.95 and well- or moderately differentiated), and a high-potential group (SUVmax < 15.95 and well- or moderately differentiated). The probability of PR-positive status in these groups was 37.5% (3/8), 72.2% (13/18), and 100.0% (36/36), respectively (P < 0.001; Table 4).

**Table 4:** Rates of positive ER and PR expression in patients with endometrial cancer with low, moderate, and high potential for ER/PR expression, as indicated by SUVmax and histologic type.

	**ER expression (%)**			**PR expression (%)**		
**Potential**	**Negative**	**Positive**	**P**	**Negative**	**Positive**	**P**
Low	62.5	37.5	<0.001	62.5	37.5	<0.001
Moderately	26.3	73.7		27.8	72.2	
High	0	100		0	100	

## DISCUSSION

Progestogens are widely used for the treatment of patients with metastatic endometrial cancers or younger patients [20–22]. The status of ER/PR in endometrial cancers is being explored as a predictive marker for response to progestogen therapy, i.e., high ER/PR expression shows a significant association with good response [8]*.* Testing for ER/PR status is now common in the management of endometrial cancer [21]. The current study found ER and PR expression rates of 83.9% and 85.5%, respectively, which were similar to those reported previously [23, 24]. ^18^F-FDG PET/CT is a noninvasive diagnostic tool to detect malignant tumors [25]. Many previous studies have suggested that ^18^F-FDG PET/CT has the potential for predicting the status of ER/PR in breast cancer [17–19]. In this study, we demonstrate that the SUVmax was significantly lower in endometrial cancer with positive ER/PR expression than in endometrial cancers that lacked ER/PR expression. This is the first study, to our knowledge, that analyzes the correlation between ^18^F-FDG accumulation and ER/PR status in endometrial cancer patients.

Hormone therapy targeting progestogens was widely used for treating many malignant tumours, including endometrial cancer [20]. However, the clinicopathologic characteristics of patients correlated with response from hormone therapy are still unknown, and identifying patients who are possible to achieve response from progestogens while excluding those who are unresponsive to the treatment is still an important question. The status of ER/PR assessed by immunohistochemistry analysis is considered as a predictive marker for progestogens treatment in endometrial cancer [21]. Whereas, tumor tissue obtained by curettage or surgical resection are invasive. Though several studies have reported the application of 16a-18F-fluoro-17b-estradiol (^18^F-FES) PET/CT in ER-positive breast cancer [26, 27], there are no studies reporting the application of ^18^F-FES PET/CT in endometrial cancers. For these reasons, other noninvasive methods, such as ^18^F-FDG PET/CT, which could predict the expression of ER/PR and inform optimal treatment decision with hormone therapy would be of important clinical value in endometrial cancers.

We discovered a negative correlation between SUVmax and ER/PR status in endometrial cancers. The ROC curves analysis demonstrated that ^18^F-FDG accumulation of primary tumors could be useful for predicting ER/PR status. Multivariate analysis revealed that both SUVmax and tumour differentiation grade were significant predictors of ER/PR expression in endometrial cancers. However, the molecular mechanism of association between ^18^F-FDG accumulation and ER/PR status are still unclear. HIF1α played a key role in regulating ^18^F-FDG accumulation of tumor cells [28, 29]. Previous studies have identified that HIF1α directly down-regulates ER expression levels in cancer lines [30–33]. In addition, Cerci et.al reported an inverse correlation between HIF-1α levels and PR expression [33, 34]. These data suggest that a negative correlation between ^18^F-FDG accumulation and ER/PR status may reflect the activation of HIF-1α pathway. We divided endometrial cancers into three groups based on their potential for being ER-positive or PR-positive, as indicated by SUVmax and tumour differentiation grade: low potential, moderate potential, and high potential. ER-positive was found in 100.0% of the endometrial cancers in the high-potential group, but only in 37.5% of endometrial cancers in the low-potential group. Similarly, PR-positive was found in 100.0% of the endometrial cancers in the high-potential group, but only in 37.5% of endometrial cancers in the low-potential group. These data demonstrate that progestogen treatment may not be recommended for endometrial cancers with low potential of being ER/PR-positive. Novel hormone therapy approaches are now being developed to target the ER/PR axis. For these reasons, noninvasive strategies, including molecular imaging tools, which could be used for predicting the status of ER/PR are of important clinical value, and have good prediction effect of the response to hormone therapy in endometrial cancers.

This study is limited by its small sample and retrospective design. Though ^18^F-FDG PET/CT could have a good predictive value, it is not feasible to obtain an optimal cutoff for SUVmax in the clinical setting, and ^18^F-FDG PET/CT cannot supersede immunohistochemistry analysis for detecting ER/PR expression. And there is a partial overlap between positive and negative ER/PR cases in ^18^F-FDG uptake. In addition, most of patients had positive ER and PR expression and this may influence the statistical analysis.

## CONCLUSIONS

Our study demonstrates that higher ^18^F-FDG accumulation in endometrial cancers is correlated with negative ER/PR expression. ^18^F-FDG PET/CT may be used to predict the status of ER/PR and thus aid in optimal treatment decision in endometrial cancers. This study can promote the advancement of noninvasive methods to infer ER/PR status. Progress in new radiotracers may improve the accuracy of this technique.

## MATERIALS AND METHODS

### Study population

Sixty-two women with endometrial cancer were examined in this study. Before ^18^F-FDG PET/CT scans were obtained, endometrial cancer was confirmed in 60 patients by using curettage, hysteroscopy, or colposcopy. Endometrial cancer was suspected in two patients but was not confirmed by pathologic tests before ^18^F-FDG PET/CT scans. All patients underwent ^18^F-FDG PET-CT before radical treatment at Ren Ji Hospital between December 2015 and April 2019. Inclusion criteria were as follows: (1) they had been treated by hysterectomy with lymphadenectomy; (2) endometrial cancers were confirmed by pathology of surgical specimens, curettage, hysteroscopy, or colposcopy; (3) adjuvant therapy had not been administered before scan; and (4) clinicopathological data were all available, including age, menopause status, biopsy method, FIGO stage, and the time from biopsy to scan, tumour size, and histologic type, were available. Informed consent was not obtained, and the RenJi Hospital Institutional Review Board approved this retrospective study.

### PET-CT

Endometrial cancer patients were asked to fast for more than six hours before ^18^F-FDG injected. Patients’ glucose levels were measured before ^18^F-FDG administration, and there were no patients whose blood glucose level exceeded 140 mg/dL in this study. The mean uptake time was approximately 60 minutes (ranged from 50 to 70 minutes). PET was carried out with an acquisition time of 3 minutes per bed position by a combined PET/CT (Biograph mCT; Siemens). The CT was used for attenuation correction.

Two board-certified nuclear medicine physicians (Ruohua Chen and Jianjun Liu) assessed the ^18^F-FDG accumulation. ROIs were placed on the tumor uptake lesion of axial section for semi-quantitative analysis. The following formula was used to calculating the maximum of standardized uptake value (SUVmax) of the primary tumor: decay-corrected tracer tissue concentration (injected ^18^F-FDG dose/patients’ weight).

### Pathological evaluation

One board-certified pathologist assessed the primary tumour. Pathological parameters were recorded, including tumour histological type, FIGO stage, maximum tumour size, depth of myometrial invasion, and pelvic or paraaortic lymph node metastasis.

### Immunohistochemical analysis

Tumor tissues were paraffin-embedded and used for immunohistochemical analysis. Positivity for ER and PR was assessed by one board-certified pathologist. The percentage of cells that stained positively for ER or PR was quantified. Cases in which more than 5% of tumour cells stained positive for ER/PR were considered to reflect positive expression [23].

### Statistical analysis

All values are demonstrated as mean ± SD. The statistical differences between different groups were compared using Mann–Whitney U test or chi-square test. P value < 0.05 was considered as significant. SPSS software was used for statistical analysis.

### Ethics approval and consent to participate

The study was approved by the institutional review board of the Shanghai Jiaotong University–affiliated Ren Ji Hospital and was in accordance with the 2013 revision of the Declaration of Helsinki. The need for informed consent was waived due to the retrospective nature of the study.
